# Omega-3 Fatty Acid and Iron Supplementation Alone, but Not in Combination, Lower Inflammation and Anemia of Infection in *Mycobacterium tuberculosis*-Infected Mice

**DOI:** 10.3390/nu12092897

**Published:** 2020-09-22

**Authors:** Arista Nienaber, Jeannine Baumgartner, Robin C. Dolman, Mumin Ozturk, Lizelle Zandberg, Frank E. A. Hayford, Frank Brombacher, Renee Blaauw, Suraj P. Parihar, Cornelius M. Smuts, Linda Malan

**Affiliations:** 1 Centre of Excellence for Nutrition, North-West University, Potchefstroom 2531, South Africa; jeannine.baumgartner@hest.ethz.ch (J.B.); Robin.Dolman@nwu.ac.za (R.C.D.); Lizelle.Zandberg@nwu.ac.za (L.Z.); feahayford220580@gmail.com (F.E.A.H.); Marius.Smuts@nwu.ac.za (C.M.S.); Linda.Malan@nwu.ac.za (L.M.); 2 Laboratory of Human Nutrition, ETH, 8092 Zurich, Switzerland; 3 International Centre for Genetic Engineering and Biotechnology (ICGEB), Cape Town-Component, University of Cape Town, Cape Town 7925, South Africa; mumin.ozturk@gmail.com (M.O.); frank.brombacher@uct.ac.za (F.B.); Suraj.Parihar@uct.ac.za (S.P.P.); 4 Institute of Infectious Diseases and Molecular Medicine (IDM), Division of Immunology and South African Medical Research Council (SAMRC) Immunology of Infectious Diseases, University of Cape Town, Cape Town 7925, South Africa; 5 Department of Nutrition and Dietetics, School of biomedical and Allied Health Sciences, College of Health Sciences, University of Ghana, Accra Box KB143, Ghana; 6 Wellcome Centre for Infectious Diseases Research in Africa (CIDRI-Africa) and Institute of Infectious Diseases and Molecular Medicine (IDM), University of Cape Town, Cape Town 7925, South Africa; 7 Division of Human Nutrition, Stellenbosch University, Tygerberg, Cape Town 7505, South Africa; rb@sun.ac.za; 8 Division of Medical Microbiology, Institute of Infectious Diseases and Molecular Medicine (IDM), Department of Pathology, Faculty of Health Sciences, University of Cape Town, Cape Town 7925, South Africa

**Keywords:** anemia of infection, docosahexaenoic acid, eicosapentaenoic acid, inflammation, iron, tuberculosis

## Abstract

Progressive inflammation and anemia are common in tuberculosis (TB) and linked to poor clinical outcomes. Eicosapentaenoic acid (EPA) and docosahexaenoic acid (DHA) have inflammation-resolving properties, whereas iron supplementation in TB may have limited efficacy and enhance bacterial growth. We investigated effects of iron and EPA/DHA supplementation, alone and in combination, on inflammation, anemia, iron status markers and clinical outcomes in *Mycobacterium tuberculosis*-infected C3HeB/FeJ mice. One week post-infection, mice received the AIN-93 diet without (control) or with supplemental iron (Fe), EPA/DHA, or Fe+EPA/DHA for 3 weeks. Mice supplemented with Fe or EPA/DHA had lower soluble transferrin receptor, ferritin and hepcidin than controls, but these effects were attenuated in Fe+EPA/DHA mice. EPA/DHA increased inflammation-resolving lipid mediators and lowered lung IL-1α, IFN-γ, plasma IL-1β, and TNF-α. Fe lowered lung IL-1α, IL-1β, plasma IL-1β, TNF-α, and IL-6. However, the cytokine-lowering effects in the lungs were attenuated with Fe+EPA/DHA. Mice supplemented with EPA/DHA had lower lung bacterial loads than controls, but this effect was attenuated in Fe+EPA/DHA mice. Thus, individually, post-infection EPA/DHA and iron supplementation lowered systemic and lung inflammation and mitigated anemia of infection in TB, but not when combined. EPA/DHA also enhanced bactericidal effects and could support inflammation resolution and management of anemia.

## 1. Introduction

Anemia is a common complication in tuberculosis (TB), affecting 30–94% of diagnosed TB patients [1,2,3,4,5,6]. In addition, anemia has been linked to poor TB outcomes, such as delayed sputum conversion, higher mortality rates and TB reoccurrence [3,7,8,9]. Exaggerated progressive inflammation is characteristic of TB, and induces hepatic hepcidin synthesis [2,10]. Hepcidin is the main regulator of iron homeostasis and mediates the degradation and internalization of the iron exporter ferroportin in macrophages, hepatocytes, and enterocytes [11]. This leads to intracellular sequestration of iron and the inhibition of iron absorption [12,13]. Consequently, hepcidin limits iron availability for pathogens that rely on host iron stores [14,15]. The persistent inflammation in TB patients also restricts iron availability for erythropoiesis and results in anemia of infection [6,16]. Anemia of inflammation (which includes anemia of infection) and iron-deficiency anemia are the two most common anemias worldwide and often co-exist [17,18]. Although less common in TB, iron-deficiency anemia resulting from low dietary iron intake, amongst other causes, also affects TB patients and may contribute to disease susceptibility and progression [1,2,3,19]. Iron supplementation may be essential for those patients with iron-deficiency anemia [1,2,3,19,20]. However, the treatment of anemia with iron is complicated by impaired absorption and the risk of promoting bacterial growth and inflammation, resulting in poor clinical outcomes [6,14].

Omega-3 long-chain polyunsaturated fatty acids (n-3 LCPUFAs) have anti-inflammatory and pro-resolving properties, and our group and others have demonstrated that these fatty acids (FAs) lower the excessive inflammation in TB [21,22]. Omega-3 LCPUFAs serve as precursors for lipid mediator (LM) synthesis, including the inflammation-resolving protectins, resolvins, and maresins that are metabolized from docosahexaenoic acid (DHA) and eicosapentaenoic acid (EPA) [23]. Therefore, we hypothesized that n-3 LCPUFA administration would result in a shift towards a more pro-resolving LM profile and a reduction in inflammation, which might lower the burden of anemia of infection in TB patients.

In addition, our group has previously shown that iron supplementation resulted in a pro-inflammatory LM profile and increased respiratory morbidity in iron-deficient South African (SA) school-children [24,25]. However, in children supplemented with a combination of iron and EPA plus DHA a pro-resolving LM profile was maintained, and the iron-induced increase in respiratory morbidity was attenuated [24,25]. We, therefore, hypothesized that providing a combination treatment of iron and n-3 LCPUFAs might potentially be an approach to deliver iron more safely in TB. Thus, this study aimed to investigate the effect of iron and EPA plus DHA supplementation, alone and in combination, on inflammation, anemia and iron status markers, as well as bacterial burden, in *Mycobacterium tuberculosis* (*Mtb*)-infected C3HeB/FeJ mice.

## 2. Materials and Methods

### 2.1. Animals and Ethics Statement

Male C3HeB/FeJ mice (Jackson Laboratory, Bar Harbour, ME, USA), aged 10 to 12 weeks, were bred and housed at the Institute of Infectious Diseases and Molecular Medicine (IDM), University of Cape Town (UCT), Cape Town, SA. Following infection, mice were housed in a biosafety level 3 containment facility, five per individually ventilated cage with filter tops (type 2 long), as well as dried wood shavings and shredded filter paper as floor coverings. The temperature range was set at 22–24 °C and 12 h–12 h light cycles. The experiments were performed following SA National Guidelines and UCT practice guidelines for laboratory animal procedures. The protocol was approved by the Animal Ethics Committee, Faculty of Health Sciences, UCT (AEC 015/040) and the AnimCare Animal Research Ethics Committee of the North-West University (NWU-00260-16-A5).

### 2.2. Experimental Design and Animal Diets

**Figure 1:**
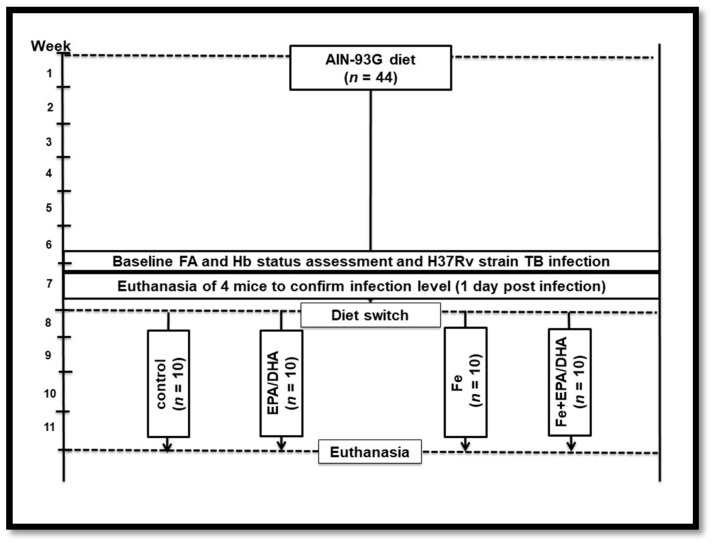
Experimental study design. EPA/DHA: eicosapentaenoic and docosahexaenoic acid supplemented group; FA: fatty acid; Fe: iron supplemented group; Fe+EPA/DHA: iron and eicosapentaenoic and docosahexaenoic acid supplemented group; Hb: hemoglobin.

Figure 1 provides an overview of the experimental design. Mice had ad libitum access to food and water and were conditioned on the same standardized AIN-93G purified rodent diet for six weeks before infection. Baseline hemoglobin (Hb) concentrations in all mice, and the total phospholipid FA composition of red blood cells (RBCs) in a sub-sample of mice (*n* = 6), were measured in tail vein blood before infection. Mice were then infected with *Mtb* via the aerosol route (described below). One week post-infection, the mice were randomly allocated to continue on the AIN-93G control diet (control, *n* = 10); to receive the AIN-93G control diet supplemented with EPA and DHA (EPA/DHA, *n* = 10); the AIN-93G control diet supplemented with iron (Fe, *n* = 10); or the AIN-93G control diet supplemented with iron and EPA plus DHA (Fe+EPA/DHA) (*n* = 10). The mice were fed these diets for three weeks, whereafter they were euthanized at 28 days post-infection. An uninfected reference group was kept on the same AIN-93G control diet throughout the study period (*n* = 3). All the purified experimental diets were obtained commercially (Dyets, Bethlehem, PA, USA), and were based on the AIN-93G formulation [26], all containing 10% fat, but with modifications in fat source and/or iron content. The dietary FA and iron composition of the experimental diets are presented in Table 1. All the diets were isocaloric with the same macronutrient content. The EPA/DHA supplemented diets (EPA/DHA and Fe+EPA/DHA) contained commercially obtained Incromega TG4030 oil (Croda Chemicals Europe, Goole, East Yorkshire, UK) with a minimum EPA at 44% of total FAs and DHA at 28% of total FAs. Gas chromatography-mass spectrometry (GCMS) analysis was performed by the manufacturer to confirm the FA and iron composition of the diets (Table 1). The daily food intake per mouse was calculated by dividing the weekly food intake by seven (days) and then by five (five mice per cage). Bodyweight and food intake of mice were measured weekly. The results of this experiment were reproduced in a second experiment (resulting in ten mice per treatment group). The data of one experiment (five mice per group) are presented in this article.

### 2.3. Aerosol Infection

A virulent *Mtb* H37Rv strain was cultured and stocks were prepared and stored at −80 °C, as described elsewhere [27]. Mice were exposed to aerosol infection for 40 min by nebulizing 6 mL of a suspension that contained 2.4 × 10^7^ live bacteria in an inhalation exposure system (model A4224, Glas-Col). One day following infection, four mice were euthanized to confirm the infection dose, which was 500 colony-forming units (CFU)/mouse.

### 2.4. Endpoint Blood and Tissue Collection

After three weeks of receiving intervention diets, mice were euthanized by halothane exposure, followed by blood collection via heart puncture. The blood was collected into ethylenediaminetetraacetic acid (EDTA) coated Microtainer^®^ tubes (K2EDTA, 1000 µL, BD), Hb measured in whole blood and then centrifuged at 8000 rpm. The plasma was removed for ferritin, soluble transferrin receptor (sTfR), hepcidin and cytokine analyses and buffy coat for FA analysis. The RBCs were washed twice with saline before storage at −80 °C and subsequent FA analysis. The liver and lung lobes were removed aseptically and weighed before preparation. The left lung lobe was homogenized in saline and 0.04% Tween-80 for the analysis of the bacillary load and lung cytokines. The right superior and post-caval lung lobes and the liver were snap-frozen in liquid nitrogen and stored at −80 °C for lung FA, LM and liver iron analyses. The right middle lobe was submerged in 10% neutral buffered formalin for histological analysis and the right inferior lobe prepared for flow cytometry.

### 2.5. Total Phospholipid FA Composition Analysis

FAs were extracted from ~20 mg lung tissue, homogenized in 10 µL phosphate-buffered saline with protease inhibitor (homogenization buffer) per 1 mg tissue, or from ~200 µL RBCs or peripheral blood mononuclear cells (PBMCs) collected as buffy coat. Lipids were extracted from each lipid pool with chloroform: methanol (2:1, *v*:*v*; containing 0.01% butylated hydroxytoluene) by a modification of the method of Folch et al. [28]. The lipid extracts were concentrated and the neutral lipids separated from the phospholipids by thin-layer chromatography (TLC) (silica gel 60 plates, Merck) and eluted with diethyl ether: petroleum ether: acetic acid (30:90:1, *v*:*v*:*v*). The lipid band containing phospholipids was removed from the TLC plate and transmethylated with methanol: sulfuric acid (95:5, *v*:*v*) at 70 °C for 2 h to form fatty acid methyl esters (FAME). FAMEs were analyzed with an Agilent Technologies 7890A gas chromatography system equipped with an Agilent Technologies 7000B triple quad mass selective detector (Agilent Technologies, Santa Clara, CA, USA) and quantification performed with Masshunter (B.06.00). Relative percentages of FAs (% *w/w*) were calculated by taking the concentration of a given FA as a percentage of the total concentration of all FAs identified in the sample.

### 2.6. Lipid Mediator Analysis

LMs in crude lung homogenates were analyzed with liquid chromatography-tandem mass spectrometry. Seventeen-hydroxy-docosahexaenoic acid (17-HDHA); 5-, 11-, 12-, 15- and 18-hydroxy-eicosapentaenoic acid (HEPE); 5-, 8-, 9-, 11-, 12-, and 15-hydroxy-eicosatetraenoic acid (HETE); prostaglandin D1 (PGD1); PGE2; PGE3 and PGD2 concentrations were measured. LMs were extracted from ~50 mg lung tissue, in 10 µL/mg homogenization buffer, with solid-phase extraction using Strata-X (Phenomenex, Torrance, CA, USA). The method was modified for Strata-XSPE columns from a previously described method [25]. Data were quantified with Masshunter B0502, using external calibration for each compound and internal standards (PGD2-d4, PGE2-d4, PGF2-d4 and 5- and 12-HETE-d8; 1000 pg of each (Cayman Chemicals, Ann Arbor, MI, USA)) to correct for losses and matrix effects.

### 2.7. Cytokine Analysis

The cytokines interleukin (IL)-1α, IL-1β, IL-2, IL-3, IL-4, IL-5, IL-6, IL-10, IL-12p70, IL-17, monocyte chemoattractant protein 1 (MCP-1), interferon-gamma (IFN-γ), tumor necrosis factor-alpha (TNF-α), chemokine ligand 3 (CCL3), granulocyte-macrophage colony-stimulating factor (GMCSF), and chemokine ligand 5 (CCL5) were measured in cell-free lung homogenates and plasma. Cytokines were analyzed using the Quansys Biosciences Q-Plex™ Mouse Cytokine Screen (West Logan, WV, USA) 16-plex array for mouse cytokines according to manufacturer instructions. Arrays were analyzed using the Q-View Imager Pro and Q-View Software.

### 2.8. Markers of Iron Status and Anemia of Infection

Hb concentrations were measured before infection in tail vein whole blood and directly after euthanasia blood collection in whole blood using a portable HemoCue^®^ Hb 201+ photometer (HemoCue AB, Angelholm, Sweden). Mice-specific enzyme-linked immunosorbent assays (ELISA) kits from ELAB Science (Houston, TX, USA) were used for the analysis of ferritin, sTfR and hepcidin concentrations in plasma. Liver iron analysis was performed on an Agilent 7900 quadrupole ICP-MS instrument in He collision mode MS at the Central Analytical Facilities, Stellenbosch University, SA. The instrument was calibrated with National Institute of Standards and Technology (NIST) traceable standards, and the accuracy of the method for iron in blood verified using the certified reference material Seronorm L2, prepared in the same way as the samples.

### 2.9. Lung Histopathology Analysis

Right middle lobes of the lungs were dissected out and fixed in 10% neutral buffered formalin. The tissue was processed using the Leica TP 1020 Processor for 24 h and subsequently embedded in paraffin wax. The Leica Sliding Microtome 2000R was used to cut 2 µm-thick sections of the embedded tissues. Three sections with 30 µm distance apart per section were cut, deparaffinized, and subsequently stained with the hematoxylin/eosin stain. The images were acquired in Nikon Eclipse 90i microscopes and analyzed with NIS-Elements AR software (Nikon Corporation, Tokyo, Japan) to determine the granulomatous area and alveolar space as a percentage of the total lung tissue [29].

### 2.10. Flow Cytometry

Single-cell suspensions of the lung tissues were prepared by cutting them into small pieces followed by incubation in Dulbecco’s Modified Eagle Media (DMEM) containing 0.18 mg/mL Collagenase Type I (Sigma, St. Louis, MO, USA), 0.02 mg/mL DNase I (Sigma, St. Louis, MO, USA) for 1 h at 37 °C under constant rotation, followed by being mechanically passed through a 100 μm and 70 μm cell strainer sequentially. Erythrocytes were lysed using RBC lysis buffer (155 mM NH_4_Cl, 12 mM NaHCO_3_, 0.1 mM EDTA). Cells were then counted and subjected to flow cytometry. Lymphoid and myeloid compartments were investigated in the lung samples of mice on various intervention diets. Antibodies used for flow cytometry analysis were as follows: CD64-PeCy7 (Clone X54-5/7.1), Ly6C-PerCPCy5.5 (Clone AL-21), CD11b-V450 (Clone M1/70), MHCII-APC (Clone M5/114.15.2), CD103-PE (Clone M290), CD11c-A700 (Clone HL3), SiglecF-APCCy7 (Clone E5-2440), Ly6G-FITC (Clone 1A8), PD-1-FITC (Clone 29F.1A12), CD4-BV510 (Clone RM4-5), CD44-PE (Clone IM7), NK1.1-APCCy7 (Clone PK136), CD3-A700 (Clone 500A2), CD62L-V450 (Clone MEL-14), CD19-PerCPCy5.5 (Clone 1D3), CD8-APC (Clone 53-6.7), and KLRG1-BV786 (Clone 2F1) purchased from BD (Biosciences, Johannesburg, SA) and eBioscience (ThermoFisher, Johannesburg, SA) [29,30].

### 2.11. Bacterial Load Determination

The bacterial loads of lungs were determined at euthanasia (28 days after infection). The left lung of each mouse was aseptically removed, weighed, homogenized and serial dilutions were plated onto DifcoTM Middlebrook 7H10 Agar (BD Biosciences, Johannesburg, SA) medium with oleic acid-albumin-dextrose-catalase (OADC) supplementation and 0.5% glycerol. The CFU were determined 21 days following incubation at 37 °C. Data are expressed as log10 CFU.

### 2.12. Statistical Analyses

Using the G*Power statistical package version 3.1.9.3, a Student’s *t*-test power analysis was done to identify significant differences in the concentrations of lung IFN-γ and bacterial load. A sample size of *n* = 4 was calculated for a two-sided alpha of 0.05 and a power of 80%. Effect sizes were estimated using the results of a preliminary study (unpublished results). Data are presented as mean ± standard error of the mean (SEM). Statistical analyses were performed using IBM SPSS statistics software (version 25; IBM Corporation). The main effects of EPA/DHA (EPA/DHA & Fe+EPA/DHA versus control & Fe) and iron (Fe & Fe+EPA/DHA versus EPA/DHA & control) supplementation, and their interaction (Fe × EPA/DHA), on all outcome variables, were analyzed by using two-way factor analysis of variance (ANOVA). Significant treatment effects in the absence of a significant interaction effect indicate additive effects of the treatments, whereas a significant interaction implies synergism or antagonism. In the presence of a significant main effect or interaction, between-group differences were examined using one-way ANOVA and the Tukey post-hoc test. For Hb, ferritin, sTfR, and hepcidin parameters, Fisher’s least significant difference post-hoc test was used. Data for ferritin and hepcidin concentrations were log-transformed before statistical analyses to improve normality. The differences in indices of iron status and anemia of infection between the infected intervention groups and the uninfected reference group were determined by the Dunnett two-sided *t*-test. A *p*-value of less than 0.05 was considered significant.

## 3. Results

### 3.1. Food Intake and Body Weight Gain

The food intake and percentage body weight gain of mice were measured weekly during the four-week infection period. There was no difference in food intake between groups (control, 3.41 ± 0.18 g; Fe, 3.38 ± 0.18 g; EPA/DHA, 3.18 ± 0.15 g; Fe+EPA/DHA, 3.36 ± 0.14 g). However, iron supplementation lowered the percentage of body weight gain in *Mtb*-infected mice (*p* < 0.001). The control (6.70 ± 0.63%) and EPA/DHA (8.12 ± 1.19%) groups had a significantly higher percentage weight gain compared with the groups supplemented with iron (Fe, 0.88 ± 1.50% and Fe+EPA/DHA, 0.16 ± 1.50%) (control vs. Fe, *p* < 0.001; control vs. Fe+EPA/DHA, *p* = 0.001; EPA/DHA vs. Fe, *p* = 0.002; and EPA/DHA vs. Fe+EPA/DHA, *p* = 0.005).

### 3.2. Total Phospholipid FA Composition of RBCs, PBMCs, and Crude Lung Homogenates

Table 2 presents the phospholipid FA composition of RBCs, PBMCs, and crude lung homogenates of all infected groups, measured at the endpoint (four weeks post-infection). From Table 2 it is evident that EPA/DHA supplementation resulted in higher phospholipid EPA, DHA, and total n-3 LCPUFAs (all *p* < 0.001). On the other hand, iron supplementation lowered EPA (*p* = 0.041 and *p* = 0.038) and total n-3 LCPUFAs (*p* = 0.046 and *p* = 0.011) in RBCs and PBMCs, as well as DHA in PBMCs (*p* = 0.008). With regards to n-6 PUFAs, EPA/DHA supplementation lowered arachidonic acid (AA), osbond acid, total n-6 LCPUFAs and total n-6/n-3 LCPUFA ratios in RBCs, PBMCs and crude lung homogenates (all *p* < 0.001, except for AA in PBMCs *p* = 0.001, AA in lungs *p* = 0.023 and total n-6 LCPUFAs in PBMCs *p* = 0.001). Iron also lowered osbond acid in RBCs and PBMCs (*p* = 0.008 and *p* = 0.025). Additionally, there were Fe × EPA/DHA interactions in RBCs and PBMCs (*p* < 0.021 and *p* = 0.005) to lower osbond acid. There was an effect of iron supplementation for higher AA (*p* < 0.001 and *p* = 0.013) and total n-6 LCPUFAs (*p* = 0.001 and *p* = 0.022) in PBMCs and lung homogenates, and n-6/n-3 LCPUFA ratios in RBCs, PBMCs and lung homogenates (*p* = 0.004, *p* < 0.001 and *p* = 0.039). Respective differences between groups are shown in Table 2.

### 3.3. Biomarkers of Iron Status and Anemia of Infection

We further evaluated indices of iron status as well as hepcidin concentrations to determine the effects of the intervention diets on biomarkers that have been linked to hypoferremia and anemia in TB. We observed a significant effect of EPA/DHA supplementation for lower Hb concentrations (*p* = 0.031), and a trend for an Fe × EPA/DHA interaction (*p* = 0.08) (Table 3). The Hb concentrations in the EPA/DHA supplemented mice did not differ from the control group but were significantly lower in the Fe+EPA/DHA (*p* = 0.009) compared with the Fe supplemented mice (Table 3). However, when compared with a non-infected reference group fed the control diet (*n* = 3), *Mtb*-infection did not result in lower Hb concentrations in control mice nor in the mice supplemented with Fe and/or EPA/DHA.

On the other hand, liver iron concentrations were significantly lowered by *Mtb*-infection in the control group compared with the healthy reference group (*p* = 0.048). There was a significant effect of iron supplementation in increasing liver iron concentrations (*p* = 0.002) and a tendency towards an antagonistic interaction between Fe × EPA/DHA (*p* = 0.090). The Fe group had significantly higher liver iron concentrations compared with the control (*p* = 0.006) and EPA/DHA (*p* = 0.023) groups, but this effect tended to be attenuated with combined Fe+EPA/DHA supplementation (Table 3).

*Mtb*-infection significantly increased sTfR, ferritin and hepcidin concentrations in the control group compared with a non-infected reference group (*p* = 0.002, *p* = 0.001, and *p* = 0.002) (Table 3). We found antagonistic Fe × EPA/DHA interactions on sTfR (*p* = 0.030), ferritin (*p* = 0.045) and hepcidin (*p* = 0.044) concentrations. The mice receiving the diets supplemented with EPA/DHA or Fe alone presented with significantly lower sTfR concentrations compared with the control group (both *p* = 0.045), but this sTfR lowering effect of EPA/DHA and iron alone was attenuated in mice supplemented with Fe+EPA/DHA (Table 3). Similarly, the mice supplemented with Fe alone had significantly lower ferritin concentrations (*p* = 0.040) and the mice supplemented with EPA/DHA tended (*p* = 0.06) to have lower concentrations than the control mice. The ferritin concentrations of the combined Fe+EPA/DHA group did not differ from any of the other groups. Additionally, the mice supplemented with EPA/DHA presented with significantly lower (*p* = 0.047) and the mice supplemented with iron tended to have lower (*p* = 0.07) hepcidin concentrations compared with the control group, with the Fe+EPA/DHA group not having significantly different hepcidin concentrations from the other groups. When compared with the non-infected reference group, sTfR, ferritin and hepcidin concentrations remained significantly higher in the Fe+EPA/DHA group (*p* = 0.027, *p* = 0.007, *p* = 0.029) but did not differ from the mice supplemented with EPA/DHA or Fe alone.

### 3.4. Tuberculosis Related Clinical Outcomes

**Figure 2:**
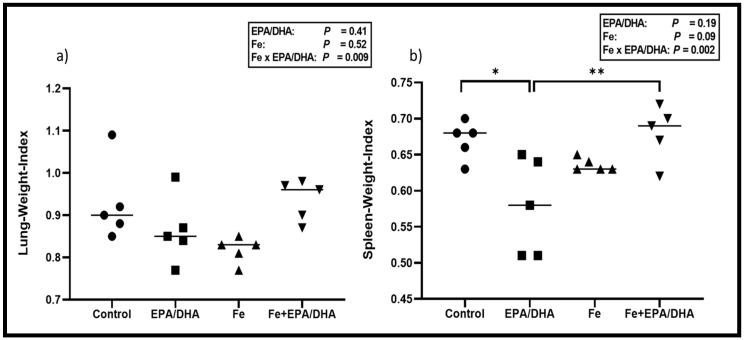
Mean (**a**) lung-weight-index and (**b**) spleen-weight-index after providing *Mtb*-infected mice with control, EPA/DHA, Fe, or Fe+EPA/DHA diets for three weeks. Results repeated in two experiments, data shown for one experiment (*n* = 5 per group). Two-way ANOVA was used to test effects of EPA/DHA (control & Fe vs. EPA/DHA & Fe+EPA/DHA), Fe (control & EPA/DHA vs. Fe & Fe+EPA/DHA), and Fe × EPA/DHA interactions. One-way ANOVA followed by Tukey post-hoc test was used to compare groups. * *p* < 0.05, ** *p* < 0.01. EPA/DHA: eicosapentaenoic and docosahexaenoic acid supplemented-group; Fe: iron-supplemented group; Fe+EPA/DHA: iron plus eicosapentaenoic and docosahexaenoic acid-supplemented group.

Figure 2 shows the lung- and spleen-weight-indexes amongst the different groups. This represents the weight of either the lungs or spleen in relation to the bodyweight of the mice (spleen or lung weight divided by endpoint bodyweight). A higher index represents worse disease severity. There were antagonistic Fe × EPA/DHA interactions on lung- and spleen-weight indexes (*p* = 0.009 and *p* = 0.002) in *Mtb*-infected mice (Figure 2). The mice supplemented with Fe alone tended to have a lower lung-weight-index when compared with the control mice (*p* = 0.08), but this lung-weight-index lowering trend of iron supplementation was attenuated in the mice supplemented with Fe+EPA/DHA (Fe+EPA/DHA vs. control, *p* = 0.99) (Figure 2a). In contrast, only the mice supplemented with EPA/DHA presented with a lower spleen-weight-index compared with the control group (*p* = 0.012), but this decrease was attenuated in the mice supplemented with Fe+EPA/DHA (Fe+DHA/EPA vs. control, *p* = 0.99) (Figure 2b).

**Figure 3:**
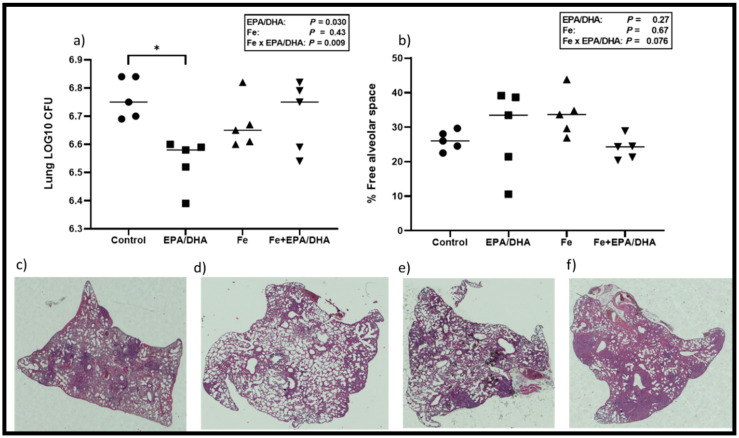
(**a**) Mean lung bacterial load, (**b**) free alveolar space (%), and representative hematoxylin-eosin staining of lungs for (**c**) control, (**d**) EPA/DHA, (**e**) Fe, (**f**) Fe+EPA/DHA groups after providing *Mtb*-infected mice with control, EPA/DHA, Fe, or Fe+EPA/DHA diets for three weeks. The values represent mean ± SEM. Results repeated in two experiments, data shown for one experiment (*n* = 5 per group). Two-way ANOVA was used to test effects of EPA/DHA (control & Fe vs. EPA/DHA & Fe+EPA/DHA), Fe (control & EPA/DHA vs. Fe & Fe+EPA/DHA), and Fe × EPA/DHA interactions. One-way ANOVA followed by Tukey post-hoc test was used to compare means. * *p* < 0.05. CFU: colony-forming units; EPA/DHA: eicosapentaenoic and docosahexaenoic acid-supplemented group; Fe: iron-supplemented group; Fe+EPA/DHA: iron plus eicosapentaenoic and docosahexaenoic acid-supplemented group.

Figure 3 shows the lung bacterial loads, percentage of free alveolar space and lung histology images, which represent the disease severity and lung pathology of the various groups. We found a significant antagonistic Fe × EPA/DHA interaction on lung bacterial load (*p* = 0.009) (Figure 3a) and a trend for an interaction on free alveolar space (%) (*p* = 0.08) (Figure 3b). Supplementation of EPA/DHA alone lowered lung bacterial load compared with the control mice (*p* = 0.008), but this effect was attenuated in the mice supplemented with EPA/DHA and Fe in combination (Fe+EPA/DHA vs. control, *p* = 0.69).

### 3.5. Lipid Mediators in Crude Lung Homogenates

**Figure 4:**
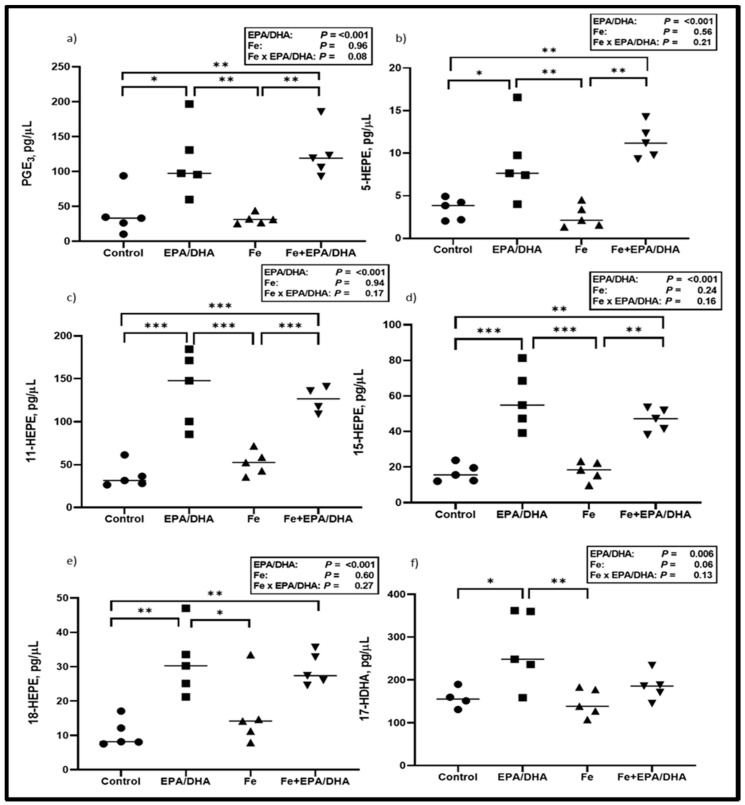
Lipid mediator concentrations including (**a**) PGE_3_, (**b**) 5-HEPE, (**c**) 11-HEPE, (**d**) 15-HEPE, (**e**) 18-HEPE and (**f**) 17-HDHA after providing *Mtb*-infected mice with control, EPA/DHA, Fe, or Fe+EPA/DHA diets for three weeks. Results repeated in two experiments, data shown for one experiment (*n* = 5 per group). Two-way ANOVA was used to test effects of EPA/DHA (control & Fe vs. EPA/DHA & Fe+EPA/DHA), Fe (control & EPA/DHA vs. Fe & Fe+EPA/DHA), and Fe × EPA/DHA interactions. One-way ANOVA followed by Tukey post-hoc test was used to compare groups. * *p* < 0.05, ** *p* < 0.01, *** *p* < 0.001. EPA/DHA: eicosapentaenoic and docosahexaenoic acid-supplemented group; Fe: iron-supplemented group; Fe+EPA/DHA: iron plus eicosapentaenoic and docosahexaenoic acid-supplemented group; HDHA: hydroxy-docosahexaenoic acid; HEPE: hydroxy-eicosapentaenoic acid.

Next, we measured lung LM concentrations, as this was the target site of our intervention and we hypothesized that LM production would be altered according to the FA composition of the intervention diets. As shown in Figure 4, EPA/DHA supplementation of *Mtb*-infected mice resulted in higher lung concentrations of the EPA-derived PGE_3_ (*p* < 0.001) and pro-resolving LM intermediates 5- (*p* = 0.047), 11- (*p* < 0.001), 12- (*p* = 0.002), 15- (*p* < 0.001), and 18-HEPE (*p* < 0.001), as well as DHA-derived 17-HDHA (*p* = 0.023) (Figure 4a–f). There was an antagonistic Fe × EPA/DHA interaction for the AA-derived pro-inflammatory intermediate 11-HETE (*p* = 0.047) and a tendency towards an antagonistic interaction for 15-HETE (*p* = 0.08), but there were no significant differences between groups (Supplemental Figure S1a,b). No treatment effects could be found on the other pro-inflammatory LMs that were measured.

### 3.6. Other Markers of the Immune and Inflammatory Response

**Figure 5:**
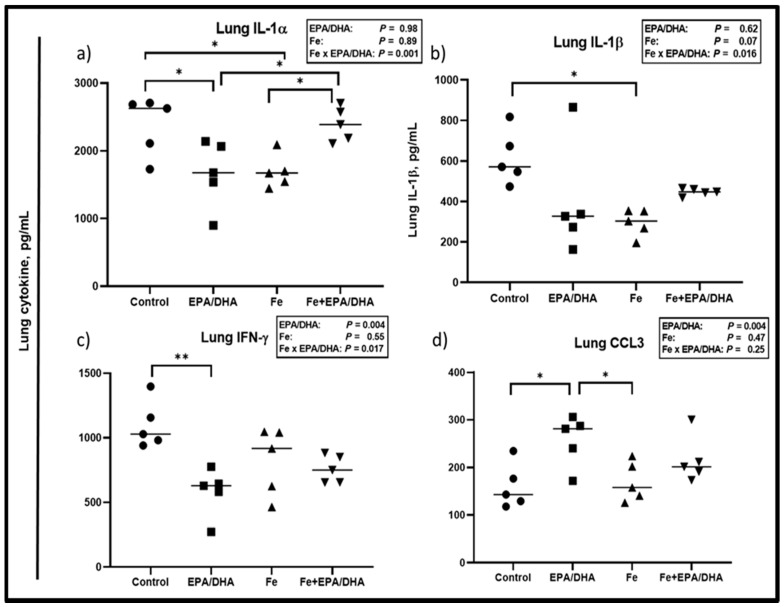
Lung cytokine concentrations after providing *Mtb*-infected mice with control, EPA/DHA, Fe, or Fe+EPA/DHA diets for three weeks, including (**a**) IL-1α, (**b**) IL-1β, (**c**) IFN-γ, and (**d**) CCL3. Results repeated in two experiments, data shown for one experiment (*n* = 5 per group). Two-way ANOVA was used to test the effects of EPA/DHA (control & Fe vs. EPA/DHA & Fe+EPA/DHA), Fe (control & EPA/DHA vs. Fe & Fe+EPA/DHA), and Fe × EPA/DHA interactions. One-way ANOVA followed by Tukey post-hoc test was used to compare means. * *p* < 0.05, ** *p* < 0.01. CCL3: chemokine ligand 3; EPA/DHA: eicosapentaenoic and docosahexaenoic acid-supplemented group; Fe: iron-supplemented group; Fe+EPA/DHA: iron plus eicosapentaenoic and docosahexaenoic acid-supplemented group; IFN-γ: interferon-gamma; IL: interleukin.

**Figure 6:**
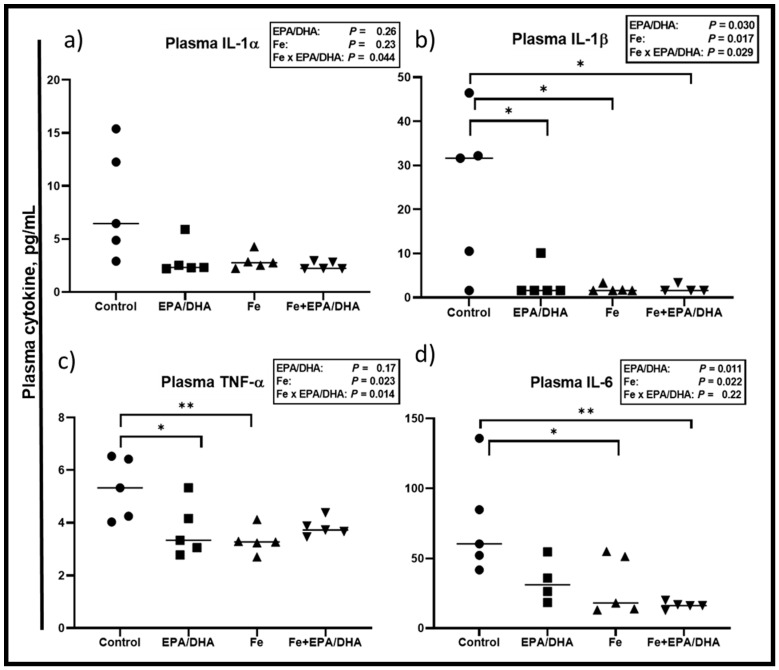
Plasma cytokine levels after providing *Mtb*-infected mice with control, EPA/DHA, Fe, or Fe+EPA/DHA diets for three weeks, including (**a**) IL-1α, (**b**) IL-1β, (**c**) TNF-α, and (**d**) IL-6. Results repeated in two experiments, data shown for one experiment (*n* = 5 per group). Two-way ANOVA was used to test effects of EPA/DHA (control & Fe vs. EPA/DHA & Fe+EPA/DHA), Fe (control & EPA/DHA vs. Fe & Fe+EPA/DHA), and Fe × EPA/DHA interactions. One-way ANOVA followed by Tukey post-hoc test was used to compare means. * *p* < 0.05, ** *p* < 0.01. EPA/DHA: eicosapentaenoic and docosahexaenoic acid-supplemented group; Fe: iron-supplemented group; Fe+EPA/DHA: iron plus eicosapentaenoic and docosahexaenoic acid-supplemented group; IL: interleukin; TNF-α: tumor necrosis factor-alpha.

Lung and plasma cytokines were measured to determine local and systemic inflammatory effects, respectively (Figure 5 and Figure 6). We observed antagonistic Fe × EPA/DHA interactions for lung IL-1α (*p* = 0.001), lL-1β (*p* = 0.003) and IFN-γ (*p* = 0.016) (Figure 5a–c). The *Mtb*-infected mice supplemented with EPA/DHA or Fe alone had significantly lower lung IL-1α concentrations than the control mice (*p* = 0.039 and *p* = 0.049), but the IL-1α-lowering effects were attenuated in mice supplemented with Fe+EPA/DHA (Figure 5a). The mice supplemented with Fe had lower lung lL-1β compared with control mice (*p* = 0.022), but not the mice supplemented with Fe+EPA/DHA (Figure 5b). Compared with the control groups, EPA/DHA supplementation significantly lowered lung IFN-γ concentrations only in the mice receiving EPA/DHA alone (*p* = 0.003), but not in the mice receiving EPA/DHA in combination with iron (Figure 5c). Furthermore, there was a main effect of EPA/DHA supplementation for higher lung CCL3 concentrations (*p* = 0.004), but only the mice receiving EPA/DHA alone had significantly higher CCL3 compared with the control mice (*p* = 0.026) (Figure 5d).

Concerning plasma cytokines (Figure 6), we observed Fe × EPA/DHA interactions for the inflammatory plasma cytokines IL-1α, IL-1β, and TNF-α (*p* = 0.044, *p* = 0.029, and *p* = 0.014) (Figure 6a–c). EPA/DHA and iron supplementation significantly and equally lowered plasma IL-1β when provided alone or in combination (*p* = 0.030, *p* = 0.007, and *p* = 0.029) (Figure 6b). A similar pattern was observed for IL-1α, TNF-α, and IL-6. There were no significant main effects or between-group differences for plasma IL-1α (Figure 6a). TNF-α was significantly lower in the mice supplemented with EPA/DHA (*p* = 0.040) and Fe (*p* = 0.009) when compared with the control mice, but only tended to be lower in the Fe+DHA/EPA group (*p* = 0.060) (Figure 6c). There were also main effects of EPA/DHA and Fe for lower plasma IL-6 concentrations (*p* = 0.022 and *p* = 0.011), resulting in significantly lower plasma IL-6 in the Fe and Fe+EPA/DHA groups compared with the control mice (*p* = 0.038 and *p* = 0.006, Figure 6d).

**Figure 7:**
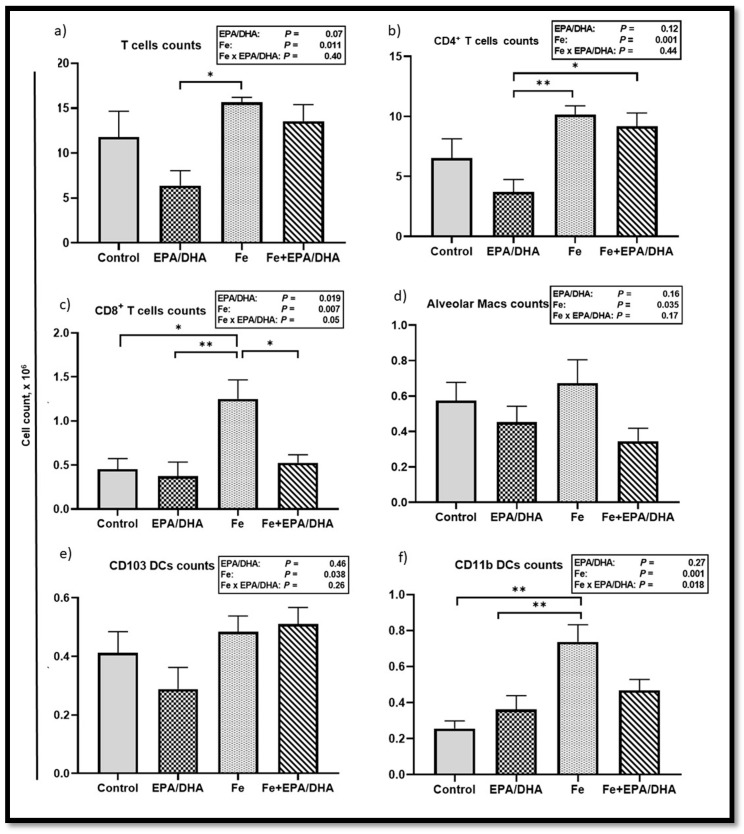
Lung immune cell counts after providing *Mtb*-infected mice with control, EPA/DHA, Fe, or Fe+EPA/DHA diets for three weeks, including (**a**) T cells, (**b**) CD4^+^ T cells, (**c**) CD8^+^ T cells, (**d**) alveolar macrophages, (**e**) CD103 DCs, and (**f**) CD11b DCs. The values represent mean ± SEM. Results repeated in two experiments, data shown for one experiment (*n* = 5 per group). Two-way ANOVA was used to test effects of EPA/DHA (control & Fe vs. EPA/DHA & Fe+EPA/DHA), Fe (control & EPA/DHA vs. Fe & Fe+EPA/DHA), and Fe × EPA/DHA interactions. One-way ANOVA followed by Tukey post hoc test was used to compare means. * *p* < 0.05, ** *p* < 0.01. DCs: dendritic cells; EPA/DHA: eicosapentaenoic and docosahexaenoic acid-supplemented group; Fe: iron-supplemented group; Fe+EPA/DHA: iron plus eicosapentaenoic and docosahexaenoic acid-supplemented group; macs: macrophages.

We also compared the lung immune cell phenotypes determined by flow cytometry and showed absolute counts (Figure 7) and percentages of total cells (Supplemental Figure S2) in a single cell suspension of the lungs. We found main effects of iron supplementation for higher counts of T cells (*p* = 0.011), CD4^+^ T cells (*p* = 0.001), CD8^+^ T cells (*p* = 0.007), interstitial macrophages (*p* = 0.004), alveolar macrophages (*p* = 0.035), CD103 dendritic cells (DCs) (*p* = 0.035), and CD11b DCs (*p* = 0.001) (Figure 7a–e, data not shown for interstitial macrophages), as well as percentage of total cells in neutrophils (*p* = 0.022), monocyte-derived DCs (mo DCs) (*p* = 0.002), natural killer (NK) cells (*p* = 0.006), T cells (*p* = 0.001), CD4^+^ T cells (*p* < 0.001), CD8^+^ T cells (*p* = 0.003), interstitial macrophages (*p* = 0.003), and CD11b DCs (*p* < 0.001) (Supplemental Figure S2a–f,h) in *Mtb*-infected mice. On the other hand, there were main effects or trends of EPA/DHA supplementation for lower T cell counts (*p* = 0.07), as well as T cells (*p* = 0.045), and CD4^+^ T cells (*p* = 0.07) as percentages of total cells. Furthermore, we found attenuating Fe × EPA/DHA interactions on CD8^+^ T cell counts (*p* = 0.050) and CD11b DC counts (*p* = 0.018) (Figure 7c,f). For both cell types, supplementation of iron alone resulted in significantly higher cell counts compared to control mice (*p* = 0.001 and *p* = 0.010), but this increase was attenuated in the Fe+EPA/DHA mice (Fe+EPA/DHA vs. control, *p* = 0.19 and *p* = 0.98).

## 4. Discussion

**Figure 8:**
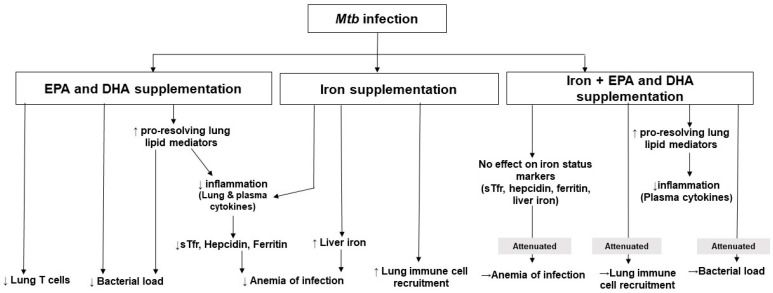
Summary of the findings of this research. DHA: docosahexaenoic acid; EPA: eicosapentaenoic acid; *Mtb: Mycobacterium tuberculosis*; sTfr: soluble transferrin receptor.

This study provides evidence, for the first time to our knowledge, that both EPA/DHA and iron independently administered to *Mtb*-infected mice, lowers inflammation and improves indices of anemia of infection. Additionally, EPA/DHA lowered bacterial load, which may be related to the enhanced phagocytic ability of immune cells [22,23] and increased synthesis of EPA and DHA-derived LMs. Interestingly, iron neither improved nor reduced bactericidal effects, but when combined with EPA/DHA supplementation, the bactericidal effect of EPA/DHA was attenuated. The main findings of this research are summarized in Figure 8.

When compared with the uninfected reference group, it was clear that the infected control mice presented with a typical profile of anemia of infection showing elevated sTfR, ferritin, and hepcidin but reduced liver iron levels [18]. Higher sTfR concentrations have been reported in inflammatory situations and in TB specifically, owing to the higher iron demand and erythropoietic stimulus resulting from inflammation-induced hypoferremia [2,18,31]. This occurrence is underpinned by the higher levels of circulating hepcidin that we found, which functions to reduce iron absorption and iron exportation from cells into plasma [18,32]. In essence, there is an intracellular shift of iron as part of the host defense to reduce iron availability for extracellular pathogens, which require iron for growth [14,33,34]. However, both EPA/DHA and iron supplementation alone lowered hepcidin levels. This may be explained by the fact that high circulating hepcidin is mainly stimulated by IL-6, but also IL-1 [32,35,36,37]. Both EPA/DHA and iron supplementation alone had a lowering effect on IL-1β and IL-6. Hepcidin is also regulated by the infection itself and has known antimicrobial properties [32,38,39]. Therefore, the finding that mice supplemented with EPA/DHA alone also had a lower bacterial burden further explains the lower hepcidin concentrations observed in this group. Additionally, sTfR concentrations increase during tissue iron deficiency but are lower where there is iron abundance, also explaining the results in the Fe group [40]. Our findings that ferritin concentrations were lower in the Fe group and tended to be lower in the EPA/DHA group compared with the control group support that ferritin instead acts as an acute-phase protein and not as a marker of stored iron in TB [6,16,41]. However, we found antagonistic interactions of iron and EPA/DHA on ferritin, TfR and hepcidin concentrations, as the lowering effect of supplementing iron or EPA/DHA individually was attenuated in mice receiving the combination treatment. Additionally, liver iron concentrations were lower in the control group when compared with the healthy reference groups, but not in the supplemented groups. Our results suggest that the provision of iron or EPA/DHA alone has similar effects of reducing inflammation and thereby improving biomarkers indicative of anemia of infection but that Fe+EPA/DHA treatment did not. Moreover, iron supplementation effectively improved iron status during infection as evident from the higher liver iron concentrations. However, Hb concentrations did not react similarly. The Hb concentrations of the infected groups were not found to be significantly different from the uninfected reference group. This was likely related to the short duration of the infection period. The erythrocyte life span in mice is ~ 40 days, which may explain why Hb concentrations remained more stable compared to other markers and may also be related to Hb’s essential biological functions [42].

Improvements in inflammation mediated the improvements in the biomarkers of iron status and anemia of infection in the EPA/DHA and Fe groups. Mutually, TNF-α, IFN-γ, and IL-1 are important in host defense against TB, but higher concentrations of these markers have also been associated with worsened lung pathology together with disease severity [43,44,45]. We found that EPA/DHA therapy lowered the pro-inflammatory plasma cytokines IL-1β and TNF-α as well as lung IL-1α and IFN-γ. This is in congruence with the lower bacterial load [45] and a more pro-resolving lung LM profile found in the EPA/DHA group, resulting from changes in the FA composition evident in all FA pools measured. In contrast to these findings, iron supplementation resulted in a higher n-6 LCPUFA composition of PBMCs and lung tissue membranes. This finding opposes previous research indicating that higher iron intakes favor higher n-3 LCPUFA membrane composition by affecting desaturase activities and/or membrane incorporation [46,47]. The reason for the discrepancies may be that these studies were conducted in healthy humans and not under infectious conditions, whereby membrane LCPUFAs may become depleted due to increased LM production during infections. Nevertheless, these FA composition changes did not influence the LM profile or cytokines in the Fe group, which presented with lower plasma IL-1β, IL-6 and TNF-α, as well as lung IL-1α and IL-1β concentrations compared with the control group. Our results agree with gene-expression studies, which found that the expression of *IL-1α*, *IL-1β*, and *TNF-α* was downregulated in macrophages, blood and livers of TB infected rabbits and mice that were supplemented with iron [48,49,50]. Similar to the EPA/DHA group, a pro-resolving LM profile was found in the Fe+EPA/DHA group, together with lower pro-inflammatory plasma cytokines. Our group had previously also established that when providing iron with EPA/DHA in iron-deficient school children, the pro-resolving LM profile produced by EPA and DHA was maintained [25].

We further investigated the effects of our intervention diets on lung immune cell phenotypes. We found that EPA/DHA lowered T cell recruitment, likely by decreasing T cell proliferation that is induced by EPA and DHA, together with the lower bacterial burden that was found in the EPA/DHA group [51,52,53]. This is supported by previous research on n-3 PUFA supplementation in TB [51]. In contrast, dietary iron supplementation led to higher counts and percentages of various immune cells in the lungs. Iron has previously been shown to affect T cell numbers, whereby iron deficiency reduces the proliferation of T cells [54,55]. The Fe group presented with higher counts of total T cells, CD4^+^ T cells, and CD8^+^ T cells. This finding is consistent with previous research, where higher CD8^+^ T cell recruitment was reported in the granulomas of *M. bovis* BCG-infected mice that received iron-rich diets [48]. When provided in combination with EPA/DHA, these effects of iron were attenuated, which was probably related to the effects of EPA/DHA described above. Furthermore, phagocytic cells are produced at infection sites to compete with the *Mtb* for iron acquisition [56,57]. This partly explains the higher macrophages, monocytes, and DCs that were evident in the lungs of mice in the Fe group, which is consistent with the findings of others [58]. However, again when supplemented with EPA/DHA, this effect of iron was attenuated. Even though there were alterations in lung inflammation and immune cell phenotypes amongst the different intervention groups, there were no main treatment effects of EPA/DHA or iron on lung pathology, which is consistent with the findings of others [48,51]. Conversely, it seemed that combination treatment again did not deliver favorable clinical outcomes, which elicited a trend to reduce free alveolar space, and therefore worsen lung pathology.

Iron supplementation did not affect bacterial load in our study. These findings, although unexpected, because iron is crucial for *Mtb* growth and virulence [34], are consistent with previous research in *Mtb*-infected animals and humans [1,48,49,59,60]. Our findings and those of previous research are partly explained by the fact that *Mtb* is an intracellular bacterium able to obtain iron, irrespective of the host iron regulatory mechanisms and iron status [1,60]. Furthermore, iron has important functions in the antibacterial activity of macrophages and T cells, and serves as co-factor for various enzymes [14,61]. This suggests its importance in host resistance, rather than promoting bacterial growth. Contrasting with our findings, others have suggested that iron abundance enhanced *Mtb* growth in mice [62,63,64], which may be related to the use of genetically modified mice [64] or intraperitoneally administered iron, resulting in high circulatory iron concentrations [62,63]. A recent review by Agoro and Mura [14] suggested an “iron benefit window” where moderate iron supplementation may enhance the immune response to lower inflammation and bacterial burden. However, beyond this threshold, iron instead augments inflammation and bacterial load [14]. Interestingly, in our study, iron lowered body weight gain in C3HeB/FeJ mice. This is in contrast with previous studies, showing no effect of iron on body weight changes in *Mtb*-infected BALB/C mice and humans and is not explained by the inflammatory profile or bacterial burden in the iron-supplemented group [1,63].

This study is strengthened by the use of a well-established TB rodent (C3HeB/FeJ) model that closely reflects human pulmonary TB pathology [65]. This is also the first study, to our knowledge, to compare iron supplementation directly with an inflammation-resolving therapy in TB. This study is limited by the fact that we measured outcomes at only one time point in the inflammatory response. Furthermore, based on its outcomes, investigating the effect of EPA/DHA as host-directed therapy adjunct to standard TB treatment in an equivalent mouse model is needed before a clinical trial can be recommended.

## 5. Conclusions

The findings of this study indicate that EPA/DHA or iron supplementation alone exerted similar benefits concerning systemic and lung inflammation and markers of anemia of infection in TB in the absence of standard TB drugs. Additionally, EPA/DHA supplementation induced a more pro-resolving lung LM profile that was maintained with combination treatment. It also enhanced bactericidal activity without influencing lung immune cell recruitment or body weight gain, as was the case with iron supplementation. However, the combination of Fe+EPA/DHA treatment did not exert the beneficial effects observed with EPA/DHA or iron supplementation alone on clinical outcomes, markers of anemia of infection, or lung inflammation. Therefore, EPA/DHA supplementation after infection may be a strategy to consider in supporting the resolution of inflammation and mitigation of anemia of infection without compromising host immunity in TB. Additionally, contrary to current belief, iron supplementation may have a modest effect on lowering inflammation without influencing lung bacterial burden. Iron supplementation may, therefore, be an effective option to improve iron status safely during TB infection, which warrants further investigation.

## Figures and Tables

**Table 1:** Iron content, fat source, and fatty acid composition of the experimental diets ^1^.

Group	Iron	Fat Source	LA	ALA	AA	DHA	EPA
	Per 100 g diet						
Control and uninfected	40 ppm	7 g soybean oil 3 g Coconut oil	3.54 g	0.44 g	<0.01 g	<0.01 g	<0.01 g
Fe	130 ppm	7 g Soybean oil 3 g Coconut oil	3.48 g	0.44 g	<0.01 g	<0.01	<0.01 g
EPA/DHA	40 ppm	7 g Soybean oil 2.7 g Coconut oil 3 g Incromega TG4030 oil	1.3 g	0.43 g	<0.01 g	0.06 g	0.09 g
Fe+EPA/DHA	123 ppm	7 g Soybean oil 2.7 g Coconut oil 3 g Incromega TG4030 oil	3.44 g	0.43 g	<0.01 g	0.06 g	0.09 g

^1^ Based on gas chromatography-mass spectrometry (GCMS) analysis of diets. AA: arachidonic acid; ALA: alpha-linolenic acid; DHA: docosahexaenoic acid; EPA: eicosapentaenoic acid; EPA/DHA: eicosapentaenoic and docosahexaenoic acid supplemented group; Fe: iron supplemented group; Fe+EPA/DHA: iron and eicosapentaenoic and docosahexaenoic acid supplemented group; LA: linoleic acid.

**Table 2:** Phospholipid FA composition of RBCs, PBMCs, and crude lung homogenates in *Mtb*-infected mice receiving control, EPA/DHA, Fe, or Fe+EPA/DHA diets for three weeks ^1^.

% Total FA	Control	EPA/DHA	Fe	Fe+EPA/DHA	*p*-Value ^2^		
					EPA/DHA	Fe	Fe × EPA/DHA
**20:5(n-3) (EPA)**							
RBC	0.13 ± 0.00 ^b^	0.51 ± 0.04 ^a^	0.12 ± 0.00 ^b^	0.43 ± 0.02 ^a^	<0.001	0.041	0.17
PBMC	0.20 ± 0.01 ^b^	0.89 ± 0.04 ^a^	0.16 ± 0.01 ^b^	0.55 ± 0.11 ^a^	<0.001	0.038	0.19
Lung	0.17 ± 0.01 ^b^	0.40 ± 0.01 ^a^	0.17 ± 0.03 ^b^	0.38 ± 0.01 ^a^	<0.001	0.74	0.63
**22:6(n-3) (DHA)**							
RBC	6.09 ± 0.21 ^b,c^	7.48 ± 0.41 ^a^	5.58 ± 0.22 ^c^	6.83 ± 0.25 ^a,b^	<0.001	0.060	0.82
PBMC	9.04 ± 0.20 ^a^	9.82 ± 0.22 ^a^	7.57 ± 0.29 ^b^	9.52 ± 0.39 ^a^	<0.001	0.008	0.060
Lung	8.08 ± 0.15 ^b^	9.68 ± 0.28 ^a^	7.99 ± 0.08 ^b^	9.86 ± 0.13 ^a^	<0.001	0.75	0.38
**Total n-3 LCPUFAs**							
RBC	6.63 ± 0.20 ^b,c^	8.59 ± 0.48 ^a^	6.06 ± 0.24 ^c^	7.80 ± 0.27 ^a,b^	<0.001	0.046	0.74
PBMC	10.60 ± 0.21 ^b^	12.40 ± 0.33 ^a^	8.76 ± 0.42 ^c^	11.80 ± 0.64 ^a,b^	<0.001	0.011	0.20
Lung	10.00 ± 0.14 ^b^	12.60 ± 0.26 ^a^	9.73 ± 0.10 ^b^	12.90 ± 0.12 ^a^	<0.001	0.96	0.07
**20:4(n-6) AA**							
RBC	17.71 ± 0.27 ^a^	16.42 ± 0.30 ^a,b^	17.68 ± 0.40 ^a^	15.88 ± 0.28 ^b^	<0.001	0.38	0.43
PBMC	16.36 ± 0.39 ^b^	14.76 ± 0.31 ^b^	20.94 ± 0.70 ^a^	17.34 ± 0.94 ^b^	0.001	<0.001	0.14
Lung	14.40 ± 0.15 ^a,b^	13.32 ± 0.42 ^b^	14.76 ± 0.27 ^a^	14.31 ± 0.23 ^a,b^	0.023	0.013	0.25
**22:5(n-6) (osbond)**							
RBC	0.87 ± 0.10 ^a^	0.37 ± 0.01 ^c^	0.49 ± 0.01 ^b^	0.35 ± 0.00 ^c^	<0.001	0.008	0.021
PBMC	1.42 ± 0.04 ^a^	0.74 ± 0.05 ^b^	1.01 ± 0.08 ^b^	0.80 ± 0.10 ^b^	<0.001	0.025	0.005
Lung	1.13 ± 0.05 ^a^	0.54 ± 0.01 ^b^	1.01 ± 0.02 ^a^	0.54 ± 0.01 ^b^	<0.001	0.27	0.050
**Total n-6 LCPUFAs**							
RBC	20.20 ± 0.32 ^a^	18.7 ± 0.44 ^a^	20.10 ± 0.47 ^a,b^	18.00 ± 0.33 ^b^	<0.001	0.33	0.45
PBMC	21.80 ± 0.41 ^b^	19.4 ± 0.54 ^b^	25.70 ± 0.78 ^a^	22.00 ± 1.16 ^b^	0.001	<0.001	0.45
Lung	21.20 ± 0.19 ^a,b^	18.3 ± 0.50 ^c^	21.50 ± 0.37 ^a^	19.90 ± 0.35 ^b^	<0.001	0.022	0.07
**Total n-6/n-3 LCPUFA ratio**							
RBC	3.05 ± 0.05 ^b^	2.19 ± 0.07 ^c,^	3.32 ± 0.07 ^a^	2.31 ± 0.04 ^c^	<0.001	0.004	0.19
PBMC	2.06 ± 0.02 ^b^	1.55 ±0.01 ^b^	2.94 ± 0.14 ^a^	1.91 ± 0.23 ^b^	<0.001	<0.001	0.07
Lung	2.12 ± 0.19 ^a^	1.45 ± 0.50 ^b^	2.21 ± 0.03 ^a^	1.54 ± 0.04 ^b^	<0.001	0.039	0.97

^1^ Values are presented as mean ± SEM. Results repeated in two experiments, data shown for one experiment (*n* = 5 per group, except for Fe group PBMCs *n* = 4 and Fe+EPA/DHA group lungs *n* = 4). ^2^ A two-way ANOVA was used to test effects of EPA/DHA (control & Fe vs. EPA/DHA & Fe+EPA/DHA), Fe (control & EPA/DHA vs. Fe & Fe+EPA/DHA), and Fe × EPA/DHA interactions. One-way ANOVA followed by Tukey post-hoc test was used to compare groups. Means in a row without common superscript letters differ significantly, *p* < 0.05. AA: arachidonic acid; DHA: docosahexaenoic acid; EPA: eicosapentaenoic acid; EPA/DHA: eicosapentaenoic acid and docosahexaenoic acid-supplemented group; FA: fatty acid; Fe: iron-supplemented group; Fe+EPA/DHA: iron plus eicosapentaenoic acid and docosahexaenoic acid-supplemented group; LCPUFA: long-chain polyunsaturated fatty acid; lung: crude lung homogenates; PBMC: peripheral blood mononuclear cells; RBC: red blood cell.

**Table 3:** Biomarkers of iron status and anemia of infection in *Mtb*-infected mice receiving control, EPA/DHA, Fe, or Fe+EPA/DHA diets for three weeks ^1^.

Iron Parameter	Non- Infected Reference (*n* = 3) ^2^	Control (*n* = 5)	EPA/DHA (*n* = 5)	Fe (*n* = 5)	Fe+ EPA/DHA (*n* = 5)	*p* Value ^3^		
						EPA/DHA	Fe	Fe × EPA/DHA
Hb (g/dL)	14.4 ± 0.8	13.6 ± 0.3 ^a,b^	13.4 ± 0.3 ^a,b^	14.3 ± 0.4 ^a^	12.9 ± 0.4 ^b^	0.031	0.59	0.08
Liver iron (µg/L)	288.0 ± 14.0	210.8 ± 12.7 ^b,c^ *	225.0 ± 16.7 ^b^	299.4 ± 15.9 ^a^	255.8 ± 18.4 ^a,b,c^	0.38	0.002	0.09
Ferritin (ng/mL)	26.1 ± 2.5	222.0 ± 43.9 ^a^ **	136.0 ± 32.5 ^a,b^	125.0 ± 23.4 ^b^	164.0 ± 13.8 ^a,b^ *	0.51	0.63	0.045
sTfR (ng/mL)	2.3 ± 0.1	17.5 ± 2.8 ^a^ **	10.8 ± 2.4 ^b^	10.8 ± 2.2 ^b^	14.5 ± 0.9 ^a,b^ **	0.49	0.50	0.030
Hepcidin (µg/mL)	0.9 ± 1.1	14.3 ± 2.7 ^a^ **	8.1 ± 2.2 ^b^	8.7 ± 2.1 ^a,b^	10.1 ± 4.6 ^a,b^ **	0.71	0.36	0.044

^1^ Values are means ± SEM. Results repeated in two experiments, data shown for one experiment (*n* = 5 per group). ^2^ Different from the uninfected reference group determined by Dunnett’s two-sided *t*-test. * *p* < 0.05, ** *p* < 0.01. ^3^ Two-way ANOVA was used to test the effects of EPA/DHA (control & Fe vs. EPA/DHA & Fe+EPA/DHA), Fe (control & EPA/DHA vs. Fe & Fe+EPA/DHA), and Fe × EPA/DHA interactions. One-way ANOVA followed by Tukey post-hoc test was used to compare groups. Means in a row without common superscript letters differ significantly, *p* < 0.05. EPA/DHA: eicosapentaenoic acid and docosahexaenoic acid supplemented-group; Fe: iron supplemented-group; Fe+EPA/DHA: iron plus eicosapentaenoic acid and docosahexaenoic acid supplemented-group; Hb: hemoglobin; sTfR: soluble transferrin receptor.

## Supplementary Materials

The following are available online at https://www.mdpi.com/2072-6643/12/9/2897/s1, Figure S1: Lipid mediator concentrations including (**a**) 11-HETE and (**b**) 15-HETE after providing *Mtb*-infected mice with control, EPA/DHA, Fe, or Fe+EPA/DHA diets for 3 weeks, Figure S2: Lung immune cells as percentages of total cells after providing *Mtb*-infected mice with control, EPA/DHA, Fe, or Fe+EPA/DHA diets for 3 weeks, including (**a**) neutrophils, (**b**) Mo dendritic cells, (**c**) natural killer cells, (**d**) T cells, (**e**) CD4^+^ T cells, (**f**) CD8^+^ T cells, (**g**) interstitial macrophages, and (**h**) CD11b dendritic cells.
