# Supplementary material for: Omega-3 Fatty Acid and Iron Supplementation Alone, but Not in Combination, Lower Inflammation and Anemia of Infection in Mycobacterium tuberculosis-Infected Mice
Source: Nutrients. 2020 Sep 22;12(9):2897. doi: 10.3390/nu12092897 (PMC7551947; doi:10.3390/nu12092897)
Supplement: Supplementary file 1 [file nutrients-12-02897-s001.pdf]

Supplement

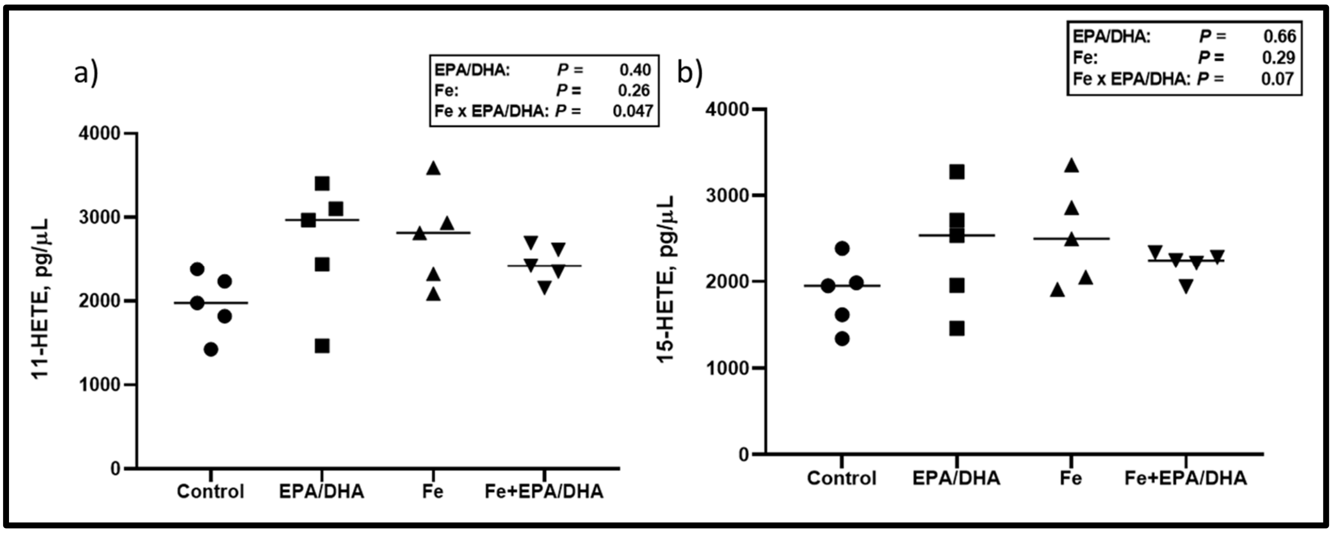

Figure S1. Lipid mediator concentrations including a) 11-HETE and b) 15-HETE after providing *Mtb*-infected mice with control, EPA/DHA, Fe, or Fe+EPA/DHA diets for 3 weeks. The values represent mean ± SEM. Results repeated in two experiments, data shown for one experiment (n=5 per group). Two-way ANOVA was used to test effects of EPA/DHA (control & Fe vs. EPA/DHA & Fe+EPA/DHA), Fe (control & EPA/DHA vs. Fe & Fe+EPA/DHA), and Fe x EPA/DHA interactions. One-way ANOVA followed by Tukey post-hoc test was used to compare groups. EPA/DHA, eicosapentaenoic and docosahexaenoic acid supplemented group; Fe, iron supplemented group; Fe+EPA/DHA, iron plus eicosapentaenoic and docosahexaenoic acid supplemented group; HETE, hydroxyeicosatetraenoic acid.

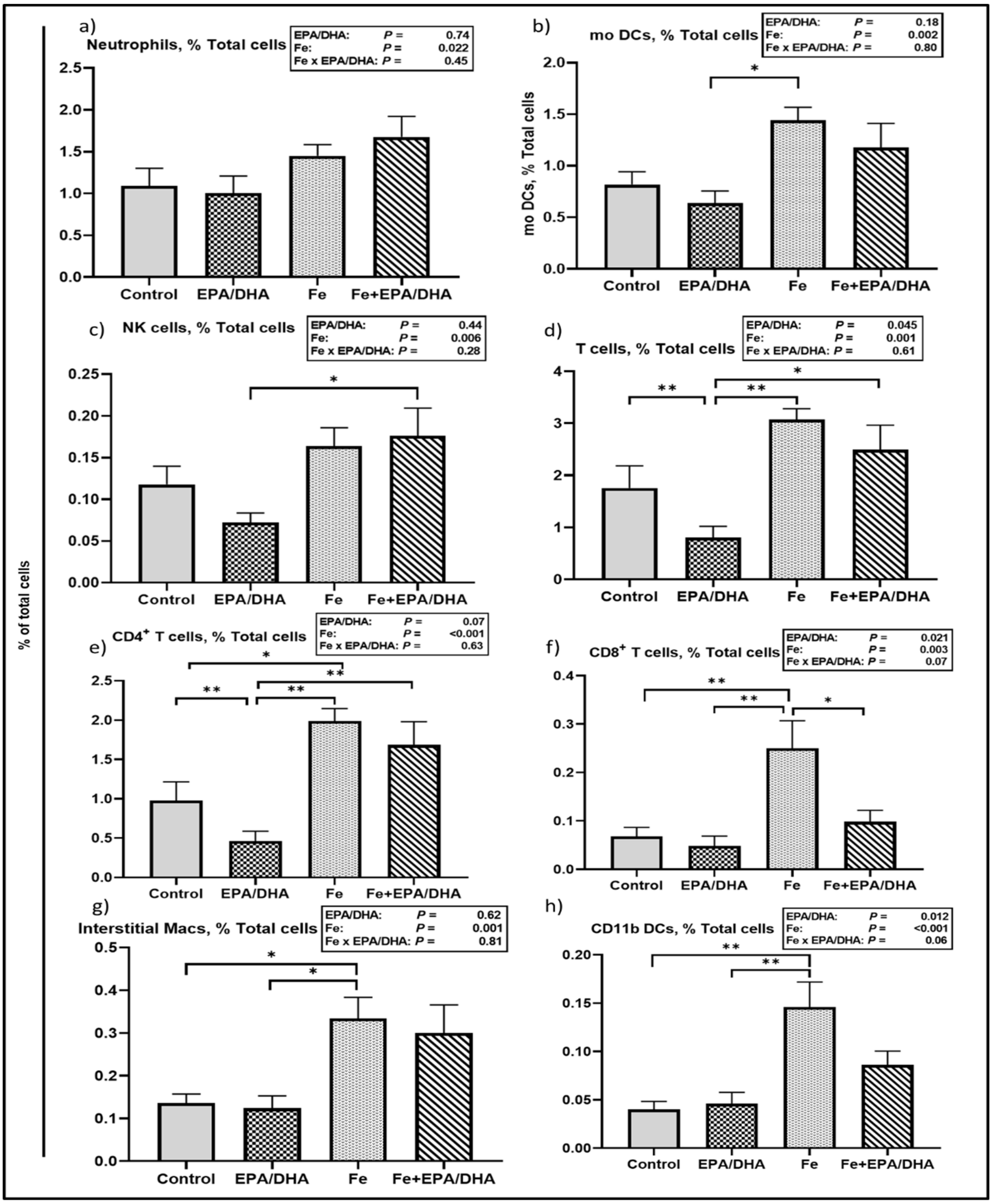

Figure S2. Lung immune cells as percentages of total cells after providing *Mtb*-infected mice with control, EPA/DHA, Fe, or Fe+EPA/DHA diets for 3 weeks, including a) neutrophils, b) Mo dendritic cells, c) natural killer cells, d) T cells, e) $CD4^+$ T cells, f) $CD8^+$ T cells, g) interstitial macrophages, and h) CD11b dendritic cells. The values represent mean ± SEM. Results repeated in two experiments, data shown for one experiment (n=5 per group). Two-way ANOVA was used to test effects of EPA/DHA (control & Fe vs. EPA/DHA & Fe+EPA/DHA), Fe (control & EPA/DHA vs. Fe & Fe+EPA/DHA), and Fe x EPA/DHA interactions. One-way ANOVA followed by Tukey post hoc test was used to compare means. *P < 0.05, **P < 0.01. DCs: dendritic cells; EPA/DHA: eicosapentaenoic and docosahexaenoic acid supplemented group; Fe: iron supplemented group; Fe+EPA/DHA: iron plus eicosapentaenoic and docosahexaenoic acid supplemented group; macs: macrophages; mo DCs: monocyte-derived DCs; NK: natural killer.
